# Sex Differences in Metabolic Recuperation After Weight Loss in High Fat Diet-Induced Obese Mice

**DOI:** 10.3389/fendo.2021.796661

**Published:** 2021-12-16

**Authors:** Santiago Guerra-Cantera, Laura M. Frago, Roberto Collado-Pérez, Sandra Canelles, Purificación Ros, Alejandra Freire-Regatillo, María Jiménez-Hernaiz, Vicente Barrios, Jesús Argente, Julie A. Chowen

**Affiliations:** ^1^ Department of Endocrinology, Hospital Infantil Universitario Niño Jesús, Instituto de Investigación La Princesa, Madrid, Spain; ^2^ Department of Pediatrics, Universidad Autónoma de Madrid, Madrid, Spain; ^3^ Centro de Investigación Biomédica en Red de Fisiopatología de la Obesidad y Nutrición (CIBEROBN), Instituto de Salud Carlos III, Madrid, Spain; ^4^ Department of Pediatrics, Hospital Universitario Puerta de Hierro-Majadahonda, Madrid, Spain; ^5^ IMDEA Food Institute, CEI UAM + CSIC, Madrid, Spain

**Keywords:** metabolism, obesity, hypothalamus, IGF1, IGF2, IGFBP2, insulin, leptin

## Abstract

Dietary intervention is a common tactic employed to curtail the current obesity epidemic. Changes in nutritional status alter metabolic hormones such as insulin or leptin, as well as the insulin-like growth factor (IGF) system, but little is known about restoration of these parameters after weight loss in obese subjects and if this differs between the sexes, especially regarding the IGF system. Here male and female mice received a high fat diet (HFD) or chow for 8 weeks, then half of the HFD mice were changed to chow (HFDCH) for 4 weeks. Both sexes gained weight (p < 0.001) and increased their energy intake (p < 0.001) and basal glycemia (p < 0.5) on the HFD, with these parameters normalizing after switching to chow but at different rates in males and females. In both sexes HFD decreased hypothalamic NPY and AgRP (p < 0.001) and increased POMC (p < 0.001) mRNA levels, with all normalizing in HFDCH mice, whereas the HFD-induced decrease in ObR did not normalize (p < 0.05). All HFD mice had abnormal glucose tolerance tests (p < 0.001), with males clearly more affected, that normalized when returned to chow. HFD increased insulin levels and HOMA index (p < 0.01) in both sexes, but only HFDCH males normalized this parameter. Returning to chow normalized the HFD-induced increase in circulating leptin (p < 0.001), total IGF1 (p < 0.001), IGF2 (p < 0.001, only in females) and IGFBP3 (p < 0.001), whereas free IGF1 levels remained elevated (p < 0.01). In males IGFBP2 decreased with HFD and normalized with chow (p < 0.001), with no changes in females. Although returning to a healthy diet improved of most metabolic parameters analyzed, fIGF1 levels remained elevated and hypothalamic ObR decreased in both sexes. Moreover, there was sex differences in both the response to HFD and the switch to chow including circulating levels of IGF2 and IGFBP2, factors previously reported to be involved in glucose metabolism. Indeed, glucose metabolism was also differentially modified in males and females, suggesting that these observations could be related.

## Introduction

The current obesity epidemic has resulted in a huge burden on healthcare systems due to its associated with an increased risk of cardiovascular diseases, type 2 diabetes (1), dyslipidemia, and certain types of cancer (2, 3), amongst other ailments and diseases. Obesity is the consequence of a positive energy balance over time (4), with caloric restriction and an improvement in dietary composition being common strategies employed to compensate for this energy excess (5). However, it is not only a challenge to lose weight, but also a problem to maintain body weight after resuming one’s normal lifestyle. A reduction in caloric intake is difficult to maintain as the resulting increase in orexigenic signals and the reduction in metabolic rate promote energy storage and weight regain (6). Whereas some metabolic parameters are restored after weight loss, others are more difficult to normalize and may require more time before doing so and could be involved in subsequent weight gain. Both nutritional status and dietary intake modify metabolic hormones, such as insulin (7) and leptin (8), which then relay these signals to central control systems in attempt to maintain metabolic homeostasis. However, less is known regarding other factors such as the insulin-like growth factor (IGF) system that is also altered according to nutritional status and dietary intake (9–11).

The IGF system includes two ligands, IGF1 and IGF2 that regulate linear growth, IGF2 mainly prenatally and IGF1 postnatally (12, 13). Although IGF2 levels are reduced postnatally it continues to exert important effects, including at the level of the brain where it is also produced (14–16). IGF1 crosses the blood-brain barrier (17) but is also locally produced by microglia and astrocytes (18, 19), oligodendrocytes (20) and neurons (21). IGF1 and 2 can bind to six different IGF-binding proteins (IGFBPs) that are able to modify their actions (22). The predominance of each IGFBP is tissue-dependent with, for example, IGFBP2 being the most abundant in the postnatal brain (23). Both IGF1 and IGF2 bind the IGF1 receptor (IGF1R), albeit with different affinities, to activate the PI3K/AKT pathway promoting cell proliferation, apoptosis inhibition, and metabolic functions (24, 25). The pappalysins, pregnancy-associated plasma protein A (PAPP-A) and PAPP-A2, cleave the binding between the IGFs and IGFBPs, allowing the free ligand to activate its receptor (26). Stanniocalcin (STC) 1 and 2 act as functional inhibitors of PAPP-A and PAPP-A2 blocking their proteolytic activity (27, 28). However, in our previous studies we did not observe modifications in levels of circulating or hypothalamic pappalysins or stanniocalcins in response to a high fat diet (HFD) (10, 11).

The hypothalamus is the main integrating center for metabolic control (29, 30). In the arcuate nucleus, neuropeptide Y (NPY) and agouti-related peptide (AgRP) neurons promote feeding behavior (31), whereas pro-opiomelanocortin (POMC) neurons promote satiety (32). Circulating metabolic hormones modulate this system, with for example ghrelin promoting NPY/AgRP neuronal activity (33) and insulin and leptin inhibiting it and activating POMC neurons (30, 34). However, little is known regarding the timing of the changes in these neuropeptides after dietary changes to stimulate weight reduction.

We previously reported that both the central and peripheral IGF systems are affected by dietary intake in a time and sex-dependent manner (10, 11). Here our objective was to determine whether peripheral and hypothalamic metabolic factors including the IGF system, are restored in HFD-induced obese C57BL/6J mice when weight-loss is induced by being placed on a normal chow diet. We also determined if there are differences between the sexes in the metabolic response to dietary change in HFD-induced obese mice. Our hypothesis is that not all metabolic parameters are normalized during the same timeframe as weight loss and that these responses are different in male and female mice.

## Material and Methods

### Ethical Statement

This study was designed and performed according to the European Communities Council Directive (2010/63/UE) and the Spanish Royal Decree 53/2013 concerning the protection of experimental animals. It was approved by the Ethical Committee of Animal Experimentation of the Hospital Puerta de Hierro de Madrid and the Animal Welfare Organ of the Comunidad Autónoma de Madrid. The number of animals used in this study was reduced to the minimum required.

### Animals and Diets

A total of 72 C57BL/6J mice (36 males and 36 females, 6-weeks old) were purchased from Charles River Laboratories. Upon arrival the animals were weighed and randomly distributed into groups of 3 mice per cage and separated by sex. They were allowed to acclimate for one week prior to being randomly assigned to a specific experimental group.

One group of mice (n = 24 for each sex) was exposed to a HFD (62% kcal from fat, 18% kcal from proteins, 20% kcal from carbohydrates, 5.1 kcal/g, LabDiet, Sodispan Research SL, Madrid, Spain) while 12 mice of each sex received standard rodent chow (6% kcal from fat, 17% kcal from proteins, 77% from carbohydrates, 3.41 kcal/g, Panlab, Barcelona, Spain). After 8 weeks, one half of the mice receiving HFD were switched to chow for 4 weeks (HFDCH) while the other half continued on the HFD. This resulted in 3 experimental groups in each sex: chow, HFD and HFDCH with 12 animals/sex/group.

### Glucose Tolerance Test (GTT)

A glucose tolerance test was performed one week prior to termination of the experiment. Six mice per group were fasted for 6 hours, weighed and then intraperitoneally injected with a D-glucose solution in PBS (0.4 g/ml) at a dose of 2 mg of D-glucose per gram of body weight. Glycemia was measured from a drop of blood from the tail by using a Freestyle Optimum Neo glucometer (Abbott, Witney, UK). Basal glycemia levels were determined just prior to the D-glucose injection (0 min), and then at 30, 60, 90 and 120 minutes after the injection.

### Fresh Tissues Collection and Sacrifices

Five days before termination of the experiment, bedding from cages of male mice was mixed into the bedding of the females’ cages to synchronize the estrous cycle. Vaginal cytology was performed in females at sacrifice, showing that 83.3% of females were in estrus and 16.7% in metestrus. Mice were weighed and then fasted 12 hours prior to sacrifice. Six mice/group were sacrificed between 9:00 and 11:00 am by decapitation. Peripheral blood was collected in tubes containing a 0.5 M ethylenediaminetetraacetic acid (EDTA) solution to prevent clotting. Tubes were then centrifuged at 3000 rpm for 15 minutes at 4 °C and plasma was aliquoted and frozen at -80 °C to avoid repeated freeze-thaw cycles. Once the brain was extracted from the skull, the hypothalamus (rostrally limited by the optic chiasm and caudally by the mammillary bodies, dorsally by the hypothalamic sulcus of Monro and thalamus, and laterally by the amygdala and the optic tracts) was isolated and frozen at -80 °C until processed.

### ELISAs

Circulating levels of free IGF1 (AnshLabs, Webster, TX, USA), total IGF1 (Mediagnost, Reutlingen, Germany), IGF2 (R&D Systems, Minneapolis, MN, USA), IGFBP2 (Millipore, Burlington, MA, USA), IGFBP3 (Mediagnost), insulin (Millipore, Burlington, MA, USA) and leptin (Millipore) were determined following the manufacturers’ instructions. Absorbance was measured by spectrophotometry (Tecan Infinite M200, Grödig, Austria). Homeostatic Model Assessment for Insulin Resistance (HOMA-IR) was calculated according to the following equation:


$$HOMA-IR=\frac{glycemia\left(\frac{mmol}{l}\right)\times insulin\left(\frac{mU}{l}\right)}{22.5}$$

### RNA and Proteins Extraction

For extraction of RNA from the hypothalamus, an RNeasy Plus Mini Kit (Qiagen, Hilden, Germany) was used according to the manufacturer’s instructions. Proteins were isolated from the eluted volume after tissue lysis. This eluate was mixed with 4 volumes of acetone and stored overnight at -20 °C. Samples were then centrifuged 10 minutes at 3000 rpm and the pellets were resuspended in a CHAPS hydrate (Sigma-Aldrich, Saint Louis, MO, USA) solution containing 7 M urea, 2 M thiourea, 4% CHAPS, 0.5% 1M Tris pH 8.8 in distilled water, being frozen at −80°C for further use. Protein Assay Dye Reagent Concentrate (Bio-Rad Laboratories, Hercules, CA, USA) was used for protein quantification by the Bradford assay.

### Western Blotting

Western blotting was performed as previously described (35, 36). Twenty µg of protein were resolved on 10 or 12% sodium dodecyl sulphate-denaturing polyacrylamide gels, transferred to polyvinylidine difluoride (PVDF) membranes that were then incubated with the primary antibody (**Table 1**) solution overnight at 4°C. The following day the membranes were washed and incubated with the appropriate secondary antibody. Peroxidase activity was detected by using Clarity Western ECL Substrate (Bio-Rad Laboratories, Hercules, California, USA), with the chemiluminescent signal being captured and quantified with ImageQuant Las 4000 Software (GE Healthcare Life Sciences, Barcelona, Spain). Glyceraldehyde 3-phosphate dehydrogenase (GAPDH) was employed as the loading control protein.

**Table 1:** Antibodies used for Western blotting.

Antibody	Isotype	Dilution	Host	Commercial source	Reference
GAPDH	Polyclonal	1:10,000	Rabbit	Sigma-Aldrich	#G9545
GFAP	Polyclonal	1:5,000	Guinea pig	Synaptic Systems	#173 004
Iba1	Polyclonal	1:1,000	Rabbit	Synaptic Systems	#234003
α-guinea pig HRP conjugated	Polyclonal	1:2,000	Goat	AbD Serotec	AHP861P
α-rabbit HRP conjugated	Polyclonal	1:20,000	Goat	Invitrogen	# 31460

### Quantitative Real-Time Polymerase Chain Reaction (RT-qPCR)

For RT-qPCR, 0.5-1 µg of RNA was retro-transcribed by using a NZY First-Strand cDNA Synthesis Kit (NZYTech, Lisbon, Portugal). TaqMan probes of the desired target genes (**Table 2**) were used and a QuantStudio 3 Real-Time PCR System (Applied Biosystems, Carlsbad, CA, USA) was employed for detection. A GAPDH endogenous control (Applied Biosystems) was chosen as the housekeeping gene. The ΔΔCT method was performed for the mathematical analysis. The results were normalized and are expressed in percentage compared to the male chow group.

**Table 2:** List of TaqMan probes used for qPCR.

Name	Gene	Reference	Commercial Source
Agouti-related protein	*Agrp*	Mm00475829_g1	Applied Biosystems
Carnitine palmitoil transferase 1a	*Cpt1a*	Mm01231183_m1	Applied Biosystems
DNA-damage inducible transcript 3 (CHOP)	*Ddit3*	Mm01135937_g1	Applied Biosystems
Fatty acid synthase	*Fasn*	Mm00662319_m1	Applied Biosystems
Insulin-like growth factor 1	*Igf1*	Mm00439560_m1	Applied Biosystems
Insulin-like growth factor 2	*Igf2*	Mm00439564_m1	Applied Biosystems
Insulin-like growth factor-binding protein 2	*Igfbp2*	Mm00492632_m1	Applied Biosystems
Interleukin-6	*Il6*	Mm00446190_m1	Applied Biosystems
Leptin receptor (ObR)	*Lepr*	Mm00440181_m1	Applied Biosystems
Neuropeptide Y	*Npy*	Mm03048253_m1	Applied Biosystems
Pro-opiomelanocortin	*Pomc*	Mm00435874_m1	Applied Biosystems
Tumor necrosis factor	*Tnf*	Mm00443260_g1	Applied Biosystems

### Statistical Analysis

Statistical analysis was performed by using SPSS 15.0 (SPSS Inc., Chicago, IL, USA) software. Differences in weight gain and food intake over time, as well as glycemia changes in the GTT, were analyzed by 2-way ANOVA with repeated measures. For the comparisons between groups, 2-way ANOVA was performed with the sex and dietary regimen (chow, HFD or HFD reverted to chow) as factors to determine if males and females were differently affected by the dietary regimens, followed by Bonferroni *post hoc* tests. If a significant difference of one factor (*i.e*., sex or diet) was found but there was no interaction between these factors, and hence the subsequent one-way ANOVA or individual t-tests are not permitted statistically, a letter indicating the overall effect of this factor was used on the graph. If there was an interaction between sex and diet, the subsequent tests, one-way ANOVA and/or t-tests) were performed with the *post-hoc* differences between individual groups represented on the graphs. A Pearson correlation coefficient was calculated for the linear correlation between variables. In all analyses p < 0.05 was considered significant.

## Results

### Body Weight and Fat Mass

**Figure 1:**
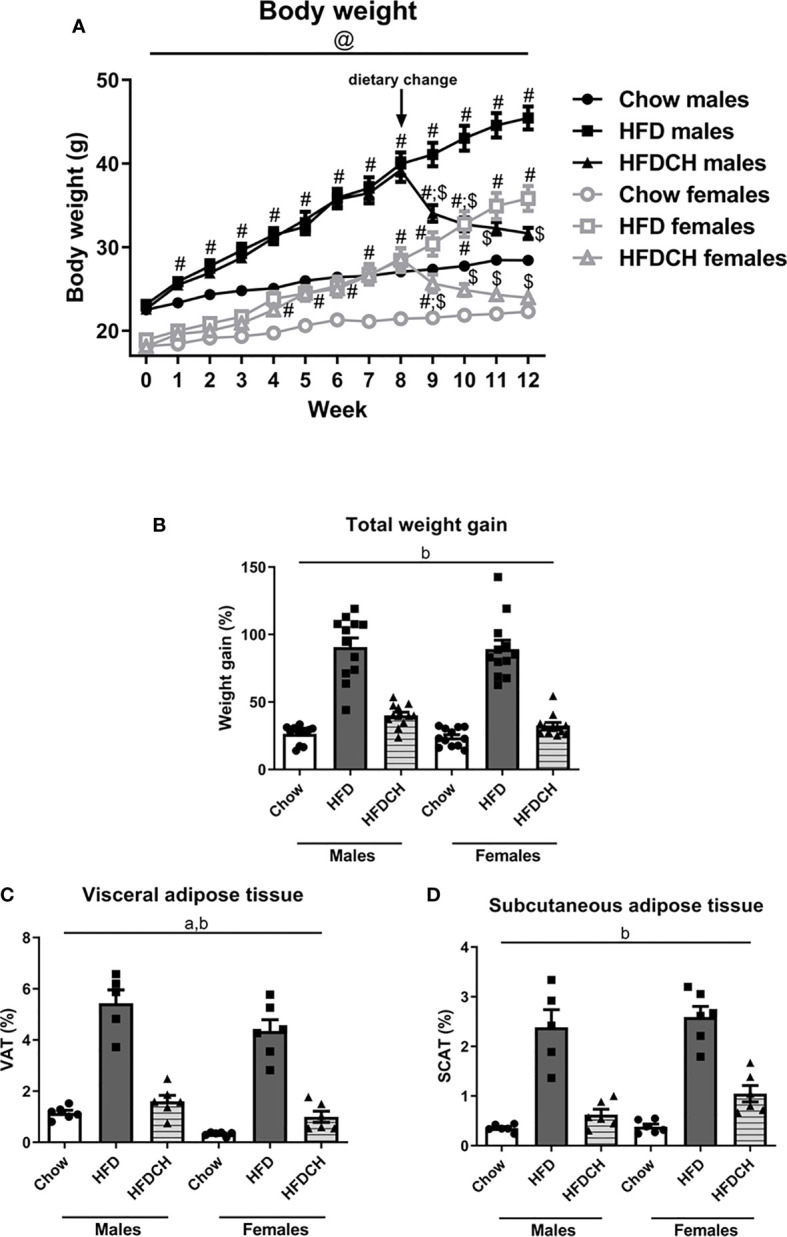
Weekly body weight progression **(A)** and total weight gain **(B)** throughout the study, as well as the percentage of visceral **(C)** and subcutaneous **(D)** adipose tissue in male and female mice exposed to a high-fat diet (HFD) or to a chow diet for 12 weeks or receiving HFD for 8 weeks and then changed to chow for the remaining 4 weeks of the study (HFDCH). a: effect of the sex. b: effect of the diet. #: different from chow in the same sex, $: different from HFD in the same sex, @, different between sexes on the same diet. n = 12.

Body weight changed throughout the study (F_(12,71)_ = 386.1, p < 0.001; **Figure 1A**), with this parameter being influenced by sex (F_(1,71)_ = 153.8, p < 0.001) and the diet consumed (F_(2,71)_ = 52.6, p < 0.001) with an interaction between sex, diet and time (F_(24,71)_ = 2.6, p < 0.001).

At study onset, there was an effect of sex on body weight (F_(1,71)_ = 249.8, p < 0.001), with males weighing more than females and this was maintained through the study. There was an overall effect of diet as early as one week after being put on the HFD (F_(2,71)_ = 13.8, p < 0.001) and this was maintained throughout the study. In males, at week 1 both the HFD and HFDCH groups weighed more than chow mice (F_(2,35)_ = 8.6, p = 0.001). In contrast, in females the increase in body weight of the HFD and HFDCH groups was not significant until week 4 (F_(2,35)_ = 11.6, p < 0.001). These differences were maintained up to week 8 in both males (F_(2,35)_ = 38.3, p < 0.001) and females (F_(2,35)_ = 5.3, p < 0.05), when the diet was changed in HFDCH mice. Final body weight was affected by sex (F_(1,70)_ = 790.1, p < 0.001; **Table 3**), with males weighing more than females, and by diet (F_(2,70)_ = 113.2, p < 0.001), with an overall increase after HFD in both sexes and no difference between the chow and HFDCH groups. Thus, the HFD-induced weight gain was more delayed in females.

**Table 3:** Final body weight, and weight gain in percentage of initial body weight up to and after the dietary change in male (M) and female (F) mice exposed to chow or a high-fat diet (HFD) for 12 weeks, or to a HFD for 8 weeks and then changed to chow for 4 weeks (HFDCH).

	Chow M	HFD M	HFDCH M	Chow F	HFD F	HFDCH F	ANOVA
Final body weight (g)	25.6 ± 0.8	42.3 ± 1.4	27.4 ± 0.5	19.3 ± 0.3	33.3 ± 1.5	21.5 ± 0.5	a, p < 0.001 b, p < 0.001
Weight gain until dietary change (%)	20.2 ± 1.5	72.6 ± 4.2 #	73.6 ± 5.9 #	18.9 ± 1.3	49.8 ± 6.0 #;@	57.7 ± 5.0 #;@	a, p < 0.001 b, p < 0.001 c, p < 0.05
Weight gain after dietary change (%)	5.2 ± 0.6	10.8 ± 3.2	-18.7 ± 2.0 #;$	4.1 ± 1.2	26.7 ± 2.3 #;@	-15.3 ± 2.4 #;$	a, p = 0.001 b, p <0.001 c, p < 0.001

a, effect of sex, b, effect of diet, c, interaction between sex and diet. #, different from chow of the same sex; $, different from HFD of the same sex; @, differences between sexes on the same diet. n = 12.

One week after returning to a chow diet, male HFDCH mice continued to weigh more than chow mice, but weighed less than the HFD group (F_(2,35)_ = 39.7, p < 0.001), with this also being observed at week 10 (F_(2,35)_ = 47.9, p < 0.001). At week 11, the HFDCH group weighed less than HFD mice and were not different from chow mice (F_(2,35)_ = 48.3, p < 0.001), with these observations continuing at week 12 (F_(2,35)_ = 54.1, p < 0.001). In females, at week 9 (one week after the diet change) HFDCH mice weighed less than HFD mice, but still weighed more than females on chow (F_(2,35)_ = 18.4, p < 0.001). From week 10 (F_(2,35)_ = 31.5, p < 0.001) to week 12 (F_(2,35)_ = 64.9, p < 0.001) female HFDCH mice weighed less than HFD mice and with no differences compared to those on chow. Thus, females returned to control levels more rapidly than males.

There was an overall effect of diet on total weight gain throughout the study (F_(2,71)_ = 135.1, p < 0.001; **Figure 1B**), being greater in mice of both sexes consuming HFD compared to those consuming only chow and those switched from HFD to chow for 4 weeks. Weight gain until the dietary change at week 8 was affected by sex (F_(1,71)_ = 13.8, p < 0.001; **Table 3**), being higher in males than females in the HFD (F_(1,23)_ = 9.9, p < 0.01) and HFDCH (F_(1,23)_ = 4.3, p < 0.05) groups. Chow-fed mice gained less weight than HFD and HFDCH mice in both males (F_(2,35)_ = 53.8, p < 0.001) and females (F_(2,35)_ = 20.2, p < 0.001), with no differences between HFD and HFDCH.

Weight gain after dietary change was affected by sex (F_(1,71)_ = 12.5, p = 0.001; **Table 3**) and diet (F_(2,71)_ = 144.5, p < 0.001), with an interaction between these factors (F_(2,71)_ = 8.7, p < 0.001). HFDCH mice reduced their weight gain after being returned to a chow diet compared to both chow and HFD in males (F_(2,35)_ = 50.9, p < 0.001) and females (F_(2,35)_ = 106.1, p < 0.001). Weight gain in HFD-fed mice was greater in females compared to males on the same diet (F_(1,23)_ = 16.6, p = 0.001).

The amount of visceral adipose tissue (VAT) as percentage of total body weight was affected by sex (F_(1,34)_ = 12.2, p < 0.01; **Figure 1C**), with males accumulating more VAT than females, and by diet (F_(2,34)_ = 114.1, p < 0.001), with an overall increase on the HFD in both sexes compared to chow and HFDCH.

Subcutaneous adipose tissue (SCAT) was globally affected by diet (F_(2,34)_ = 79.1, p < 0.001), being increased after long-term HFD consumption in both sexes (**Figure 1D**).

### Energy Intake

**Figure 2:**
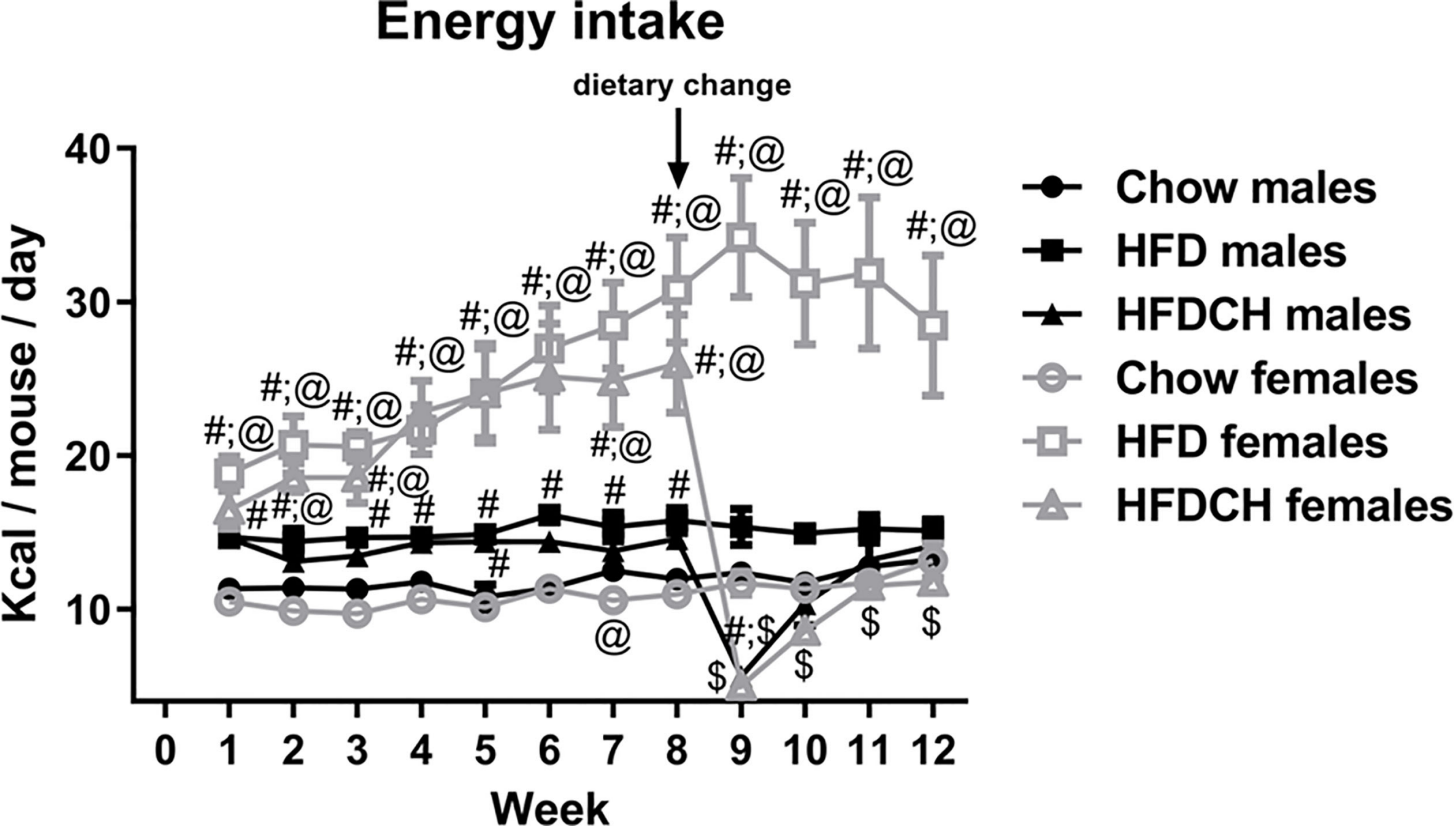
Weekly energy intake throughout the study in male and female mice that were exposed to a chow diet or high-fat diet (HFD) for 12 weeks, or to 8 weeks of HFD and then switched to chow during the last 4 weeks (HFDCH) of the study. #: different from chow diet of the same sex, $: different from HFD of the same sex, @: different between sexes on the same diet. n = 4 (number of cages per experimental group).

Energy intake throughout the study (**Figure 2**) was affected by time (F_(11,23)_ = 10.1, p < 0.001), sex (F_(1,23)_ = 58.3, p < 0.001) and dietary regimen (F_(2,23)_ = 62.5, p < 0.001) with an interaction between time, sex and diet (F_(11,23)_ = 5.1, p < 0.001), as well as sex and dietary regimen (F_(2,23)_ = 28.8, p < 0.001).

In males, energy intake was higher in the HFD and HFDCH groups compared to chow during the first week of the study (F_(2,11)_ = 15.3, p = 0.001) and also from week 4 (F_(2,11)_ = 6.8, p < 0.05) to 8 (F_(2,11)_ = 10.8, p < 0.01). One week after the dietary change, energy intake in the HFDCH group was reduced compared to both chow and HFD mice (F_(2,11)_ = 38.2, p < 0.001), with the HFD group continuing to consume more kcal than the chow group. At week 10, energy intake in the HFDCH group remained reduced compared to HFD (F_(2,11)_ = 7.6, p < 0.05), with no observed differences at weeks 11 or 12 between any of the groups. In females, HFD consumption led to increased energy intake from the first (F_(2,11)_ = 19.5, p = 0.001) to the last (F_(2,11)_ = 11.6, p < 0.01) week of the study. In the HFDCH group this difference was observed from week 1 (F_(2,11)_ = 19.5, p = 0.001) to week 8 (F_(2,11)_ = 14.4, p < 0.01), but HFDCH mice consumed fewer calories than the chow group at week 9 (F_(2,11)_ = 44.4, p < 0.01), with no differences observed between chow and HFDCH from week 10 to week 12. Thus, on a HFD males normalized energy intake more rapidly than females.

The total number of kilocalories consumed per mouse throughout the study was affected by sex (F_(1,23)_ = 35.4, p < 0.001; **Table 4**), with females consuming more kilocalories than males when on the HFD (F_(1,7)_ = 28.7, p < 0.01) or HFDCH regimen (F_(1,7)_ = 12.4, p < 0.05). The kilocalories consumed were also altered by diet (F_(2,23)_ = 39.5, p < 0.001), with an interaction between sex and diet (F_(2,23)_ = 17.4, p < 0.001). Total mean energy consumed was higher on the HFD compared to both the chow and HFDCH groups in males (F_(2,11)_ = 18.8, p = 0.01) and females (F_(2,11)_ = 29.4, p < 0.001), with HFDCH female mice consuming more total kilocalories than those on the chow diet.

**Table 4:** Energy intake parameters in male (M) and female (F) mice exposed to chow or to a high-fat diet (HFD) for 12 weeks, or 8 weeks of HFD intake and changed to a chow diet for 4 weeks (HFDCH).

	Chow M	HFD M	HFDCH M	Chow F	HFD F	HFDCH F	Significance
Total kcal/mouse	999.4 ± 26.7	1261.8 ± 21.3 #	1092.0 ± 40.7 $	922.1 ± 30.0	2224.5 ± 178.3 #;@	1493.1 ± 106.3 #;$;@	p < 0.001
Total kcal/mouse/ 100 g	3808.5 ± 77.4	3638.0 ± 149.9	3454.6 ± 174.4	4438.9 ± 124.2 @	8296.3 ± 710.4 #;@	5977.6 ± 495.0 $;@	p < 0.001
Kcal/mouse before change	648.5 ± 19.4	837.3 ± 23.5 #	788.8 ± 23.1 #	586.8 ± 12.4 @	1344.3 ± 72.6 #;@	1235.1 ± 112.0 #;@	p < 0.001
Kcal/mouse after diet change	350.9 ± 7.9	424.6 ± 19.6	303.2 ± 26.3 $	335.3 ± 18.8	880.2 ± 116.4 #;@	258.0 ± 12.2 $	p < 0.001
Total energy efficiency	0.36 ± 0.07	1.48 ± 0.07 #	0.44 ± 0.04 $	0.12 ± 0.00 @	0.67 ± 0.09 #;@	0.22 ± 0.01 $;@	p < 0.001
Energy efficiency before change	0.58 ± 0.11	1.68 ± 0.15 #	1.74 ± 0.26 #	0.53 ± 0.01	0.64 ± 0.11 @	0.73 ± 0.09 @	p < 0.001
Energy efficiency after change	0.46 ± 0.01	1.17 ± 0.18	-2.45 ± 0.87	0.21 ± 0.08	0.93 ± 0.06	-1.49 ± 0.29	b, p < 0.001

b, effect of the diet. #, different from chow in the same sex; $, different from HFD in the same sex; @, differences between sexes on the same diet. n = 4.

Energy consumption before the diet change was higher in females than males on a HFD (F_(1,7)_ = 44.2, p = 0.001; **Table 4**) and HFDCH (F_(1,7)_ = 15.2, p < 0.01), but higher in males on chow than in females on the same diet (F_(1,7)_ = 7.2, p < 0.05). Both HFD and HFDCH mice consumed more calories than those on chow in males (F_(2,11)_ = 19.7, p = 0.001) and females (F_(2,11)_ = 28.0, p < 0.001). Energy intake after dietary change was higher in females on a HFD than males on the same diet (F_(1,7)_ = 14.9, p < 0.01) during the last 4 weeks of the study. Kilocalorie intake in males during this period was higher on HFD compared to HFDCH (F_(2,11)_ = 9.9, p < 0.01) but not to chow mice, whereas HFD females consumed more energy than both chow and HFDCH females (F_(2,11)_ = 24.5, p < 0.001).

The overall energy intake per 100 grams of body weight was higher in females than males (F_(1,23)_ = 74.1, p < 0.001; **Table 4**) when exposed to chow (F_(1,7)_ = 18.6, p < 0.01), HFD (F_(1,7)_ = 41.2, p = 0.001), or the HFDCH regimen (F_(1,7)_ = 23.1, p < 0.01). Dietary consumption also affected this parameter (F_(2,23)_ = 12.9, p < 0.001), with an interaction between sex and diet (F_(2,23)_ = 14.8, p < 0.001). In females, energy intake normalized to body weight was higher in HFD compared to both chow and HFDCH (F_(2,11)_ = 14.8, p = 0.001).

Total energy efficiency, expressed as the weight gained in grams per calories consumed, was higher in males than females (F_(1,23)_ = 89.9, p < 0.001; **Table 4**) when exposed to chow (F_(1,7)_ = 12.7, p < 0.05), HFD (F_(1.7)_ = 54.8, p < 0.001) or the HFDCH regimen (F_(1,7)_ = 35.3, p = 0.001). Overall energy efficiency was higher in HFD compared to both chow and HFDCH in males (F_(2,11)_ = 112.7, p < 0.001) and females (F_(2,11)_ = 34.3, p < 0.001). In the first 8 weeks of the study, energy efficiency was higher HFD (F_(1,7)_ = 31.6, p = 0.001) and HFDCH (F_(1,7)_ = 13.5, p = 0.01) males compared to females. Energy efficiency during this period was higher in males on a HFD or HFDCH compared to chow (F_(2,11)_ = 13.0, p < 0.01), with no differences in females. After dietary change, energy efficiency was affected by diet (F_(2,23)_ = 33.8, p < 0.001), being higher on the HFD and decreased in the HFDCH group compared to the chow group in both sexes. Energy efficiency was globally affected by the dietary regimen (F_(2,23)_ = 33.8, p < 0.001; **Table 4**), being reduced in the HFDCH group compared to chow and HFD in both sexes, with no differences between chow and HFD.

### Glucose Metabolism

**Figure 3:**
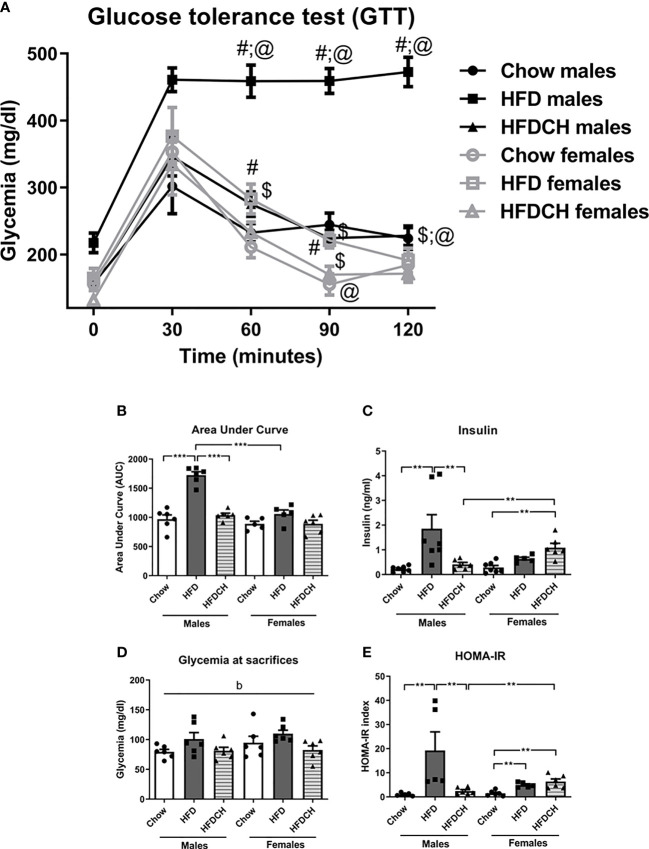
Glucose tolerance test **(A)**, the area under curve (AUC) of the GTT **(B)**, circulating insulin levels **(C)** and glycemia at sacrifice **(D)**, and Homeostatic Model Assessment for Insulin Resistance (HOMA-IR; **E**) in male and female mice exposed to chow or a high-fat diet (HFD) for 12 weeks, or HFD for 8 weeks and then switched to chow during the last 4 weeks (HFDCH) of the study. **p < 0.01, *** p< 0.001; #, different from chow, $, different from HFD, @, differences between sexes on the same diet. b: effect of the diet. n = 6.

In the GTT, glycemia changed over time (F_(4,33)_ = 88.9, p < 0.001; **Figure 3A**) with an interaction between time, sex and dietary regimen (F_(4,33)_ = 2.9, p < 0.01). There was also an effect of the sex (F_(1,33)_ = 52.0, p < 0.001) and dietary regimen (F_(2,33)_ = 45.4, p < 0.001), with an interaction between these factors (F_(2,33)_ = 19.3, p < 0.001).

Basal glycemia was affected by sex (F_(1,33)_ = 8.4, p < 0.01), with males having higher levels than females, and was increased in the HFD group (F_(2,33)_ = 9.0, p < 0.01). At 30 min there was an increase in glycemia in all groups, which was greater in the HFD group of both sexes (F_(2,33)_ = 4.0, p < 0.05). At 60 minutes, HFD males had a higher glycemia compared to both chow and HFDCH males (F_(2,17)_ = 39.0, p <0.001), whereas in females this differences was only found between the HFD and chow groups (F_(2,15)_ = 3.9, p < 0.05). HFD-exposed male mice also had a higher glycemia 60 minutes post injection compared to females on the same diet (F_(1,10)_ = 27.6, p = 0.001).

At 90 minutes, males had a higher glycemia than females when on a chow diet (F_(1,10)_ = 13.7, p < 0.01), HFD (F_(1,10)_ = 105.8, p < 0.001) or HFDCH regimen (F_(1,11)_ = 9.9, p < 0.05). The HFD group had higher glycemia compared to chow and HFDCH groups in both males (F_(2,17)_ = 63.3, p < 0.001) and females (F_(2,15)_ = 6.3, p < 0.05). At 120 minutes post injection males had higher glycemia than females in the HFD (F_(1,10)_ = 90.9, p < 0.001) and HFDCH (F_(1,11)_ = 8.3, p < 0.05) groups. HFD males had higher glycemia compared to both the chow and HFDCH (F_(2,17)_ = 62.7, p < 0.001) males, with no differences in females at this time point.

The area under the curve (AUC, **Figure 3B**) was influenced by sex (F_(1,33)_ = 38.2, p < 0.001), with males having a higher value than females, and diet (F_(2,33)_ = 37.1, p < 0.001), with an interaction between these factors (F_(2,33)_ = 14.3, p < 0.001). In the male HFD group the AUC was greater than in the chow and HFDCH groups (F_(2,17)_ = 53.1, p < 0.001), as well as compared to the female HFD group (F_(1,10)_ = 54.2, p < 0.001). There were no differences amongst the female groups.

Plasma insulin levels at sacrifice were affected by diet (F_(2,37)_ = 6.7, p < 0.01; **Figure 3C**), with an interaction between sex and diet (F_(2,37)_ = 5.9, p < 0.01). In males, the HFD group had higher insulin levels compared to the chow and HFDCH groups (F_(2,19)_ = 6.7, p < 0.01). In females, the HFDCH group had higher plasma insulin levels compared to those on the chow diet (F_(2,17)_ = 12.3, p < 0.01). Females on the HFDCH regimen also had higher insulin levels than males on the same dietary regimen (F_(1,11)_ = 12.5, p < 0.01). Glycemia levels at sacrifice were globally affected by dietary regimen (F_(2,34)_ = 4.6, p < 0.05; **Figure 3D**), being increased after the long-term HFD intake and with no differences between the chow and HFDCH groups.

The HOMA-IR index was affected by diet (F_(2,32)_ = 7.0, p < 0.01; **Figure 3E**), with an interaction between sex and diet (F_(2,32)_ = 5.4, p < 0.05). In male HFD mice the HOMA-IR index was increased compared to both chow and HFDCH mice (F_(2,15)_ = 5.7, p < 0.05). In contrast, in females the HOMA-IR index was increased in both the HFD and HFDCH groups compared to those on chow (F_(2,16)_ = 11.0, p < 0.01). Female HFDCH mice had a higher HOMA-IR index compared to males on the same dietary regimen (F_(1,11)_ = 9.6, p < 0.05).

### Plasma Levels of Leptin and Members of the IGF System

**Figure 4:**
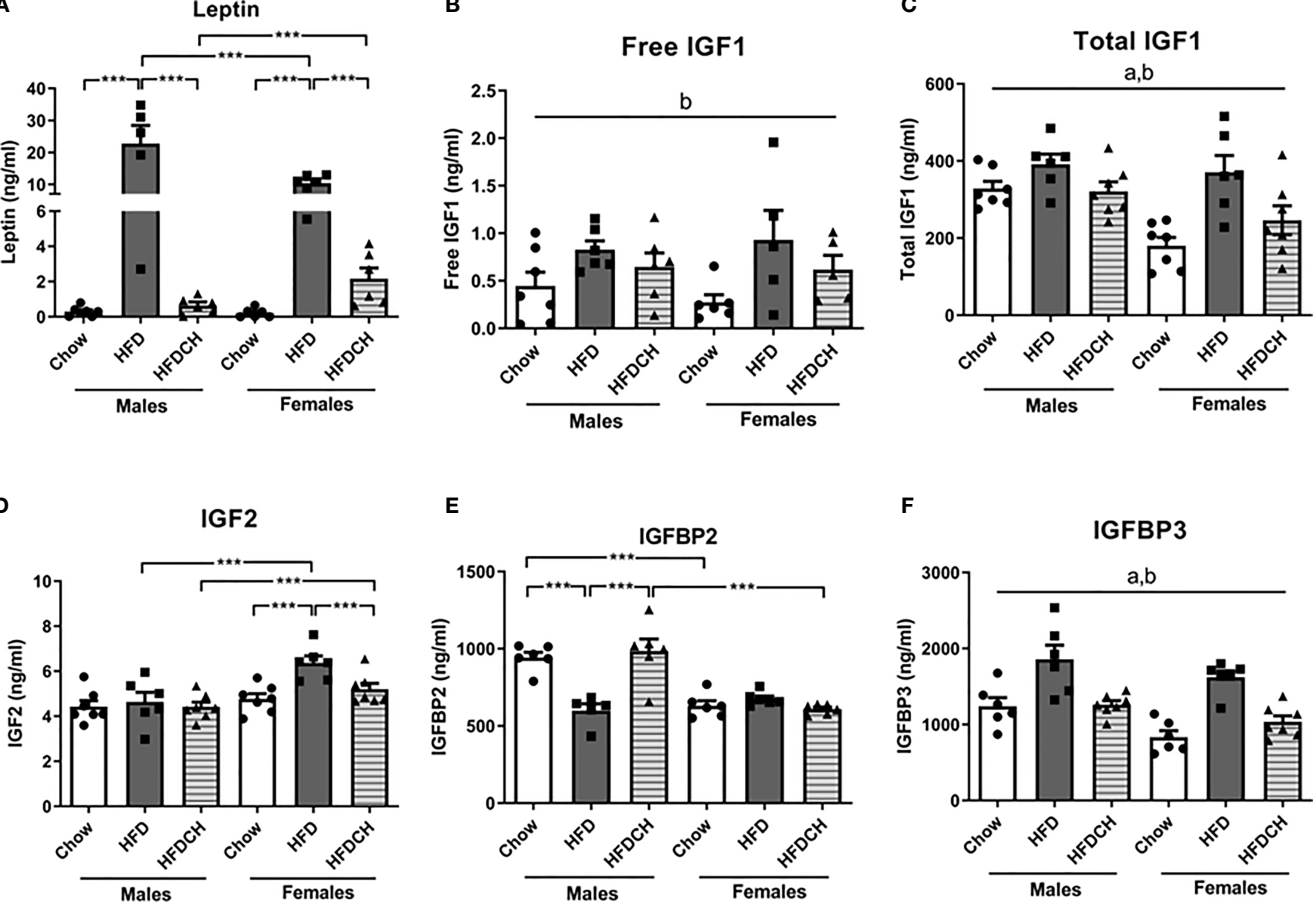
Circulating levels of leptin **(A)**, free insulin-like growth factor (IGF)1 **(B)**, total IGF1 **(C)**, IGF2 **(D)**, IGF binding protein (IGFBP)2 **(E)** and IGFBP3 **(F)** in mice that received chow or a high-fat diet (HFD) for 12 weeks, or 8 weeks of HFD and then chow during the last 4 weeks (HFDCH) of the study. ***p < 0.001, a, effect of the sex; b, effect of the diet. n = 6.

Circulating leptin levels were affected by sex (F_(1,35)_ = 5.2, p < 0.05; **Figure 4A**), with males having higher levels than females, and by diet (F_(2,35)_ = 41.1, p < 0.001) with an interaction between these factors (F_(2,35)_ = 7.2, p < 0.01). In HFD mice leptin levels were higher compared to the chow and HFDCH groups in males (F_(2,17)_ = 21.1, p < 0.001) and females (F_(2,17)_ = 51.6, p < 0.001). In HFD mice, males had higher leptin levels than females (F_(1,10)_ = 5.6, p < 0.05). In contrast, on the HFDCH regimen females had higher leptin levels compared to males (F_(1,11)_ = 5.6, p < 0.05).

Free IGF1 levels were affected by diet (F_(2,34)_ = 5.4, p = 0.01; **Figure 4B**), with an overall increase in the HFD and HFDCH groups. Total IGF1 levels were modified by sex (F_(1,39)_ = 11.5, p < 0.01; **Figure 4C**), with males having higher levels than females, and diet (F_(2,39)_ = 9.7, p < 0.001), with HFD mice having overall higher levels than both chow and HFDCH mice in both sexes.

Plasma IGF2 levels were affected by sex (F_(1,39)_ = 16.7, p < 0.001; **Figure 4D**), with females having overall higher levels compared to males with this being significant on the HFD (F_(1,11)_ = 10.8, p < 0.01) and HFDCH (F_(1,13)_ = 5.4, p < 0.05) dietary regimens. Dietary intake also modified IGF2 levels in circulation (F_(2,39)_ = 5.2, p = 0.01), with an increase in IGF2 levels in HFD females compared to both the chow and HFDCH groups (F_(2,19)_ = 9.3, p < 0.01), with no dietary effect found in males.

Circulating IGFBP2 levels were modified by sex (F_(1,34)_ = 34.8, p < 0.001; **Figure 4E**) and diet (F_(2,34)_ = 8.6, p < 0.01), with an interaction between sex and diet (F_(2,34)_ = 16.1, p < 0.001). HFD males had lower IGFBP2 levels compared to the chow and HFDCH males (F_(2,16)_ = 12.9, p < 0.01). There was no effect of diet in females. Moreover, males had higher IGFBP2 levels than females in the chow (F_(1,11)_ = 44.9, p < 0.001) and HFDCH (F_(1,11)_ = 22.3, p < 0.01) groups. Plasma IGFBP3 levels were affected by sex (F_(1,37)_ = 11.5, p < 0.01; **Figure 4F**), with males having higher levels than females, and by diet (F_(2,37)_ = 25.1, p < 0.001), with levels increasing in HFD mice and tending to normalize in HFDCH mice in both sexes.

Taking into consideration that IGFBP2 is a leptin-regulated antidiabetic factor that protects against obesity (37, 38), we calculated linear correlations between this factor in circulation with insulin, glycemia, leptin, body weight and the percentage of VAT (**Table 5**). In addition, taking into account our previous data (11) and that IGFBP2 has a higher affinity for IGF2 compared to IGF1 (39), we also calculated these correlations with IGF2. In males, IGFBP2 was negatively correlated with peripheral insulin, leptin and glycemia levels as well as with body weight and the amount of VAT. However, in females IGFBP2 was positively correlated with glycemia and VAT, but with no correlation with insulin or leptin. IGF2 was positively correlated with insulin and glycemia in males whereas in females it was positively correlated with leptin, body weight and VAT. When split by sex and diet, IGF2 was positively correlated with insulin (r = 0.827, p < 0.05) and glycemia (r = 0.852, p < 0.05) in HFD males, whereas it was negatively correlated with insulin (r = -0.850, p < 0.05) in HFDCH females. After separating by diet, but analyzing both sexes together, IGFBP2 was negatively correlated with insulin in HFD (r = -0.739, p < 0.05) and HFDCH (r = -0.659, p < 0.05) mice, whereas IGF2 showed a positive correlation with glycemia in HFD animals (r = 0.692, p < 0.05).

**Table 5:** Linear correlation between circulating levels of IGF2 and IGFBP2 with peripheral insulin, glycemia, and leptin, and with body weight and visceral adipose tissue in percentage separated by sex (M, males; F, females).

	Insulin	Glycemia	Leptin	Body weight	VAT (%)
**IGFBP2 (M)**	-0.668**	-0.494*	-0.773**	-0.703**	-0.668**
**IGFBP2 (F)**	-0.263	0.517*	0.347	0.398	0.499*
**IGF2 (M)**	0.532*	0.540*	0.353	0.366	0.329
**IGF2 (F)**	0.077	0.378	0.698**	0.621**	0.607**

*p < 0.05, **p < 0.01. n = 6.

### Hypothalamic Factors

**Figure 5:**
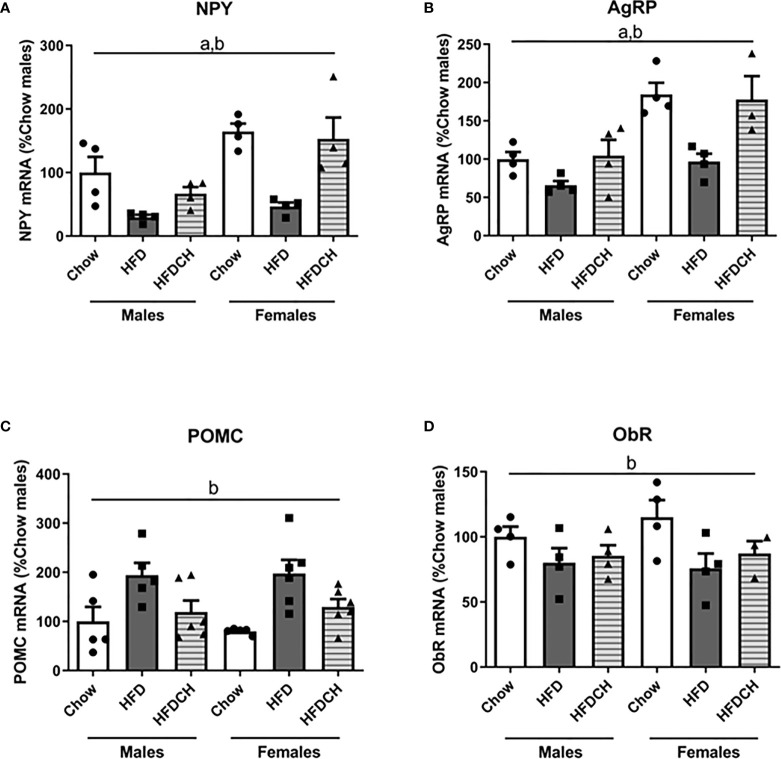
Relative mRNA levels of orexigenic and anorexigenic neuropeptides in the hypothalamus. Neuropeptide Y [NPY; **(A)**], Agouti-related protein [AgRP; **(B)**], and proopiomelanocortin [POMC; **(C)**], as well as leptin receptor [ObR; **(D)**] in mice fed chow or a high-fat diet (HFD) for 12 weeks or HFD for 8 weeks followed by chow for 4 weeks (HFDCH) 4 weeks. a, effect of the sex; b, effect of the diet. n = 4 - 6.

Relative NPY mRNA levels were determined by sex (F_(1,23)_ = 13.9, p < 0.01; **Figure 5A**), with females having higher levels than males, and diet (F_(2,23)_ = 14.3, p < 0.001). In both sexes HFD mice had lower NPY mRNA levels compared to both the chow and HFDCH groups. A similar pattern was found in AgRP mRNA levels as there was an effect of sex (F_(1,22)_ = 23.7, p < 0.001; **Figure 5B**), with females having higher levels than males, and by diet (F_(2,22)_ = 10.0, p = 0.001), with the HFD group having reduced levels in both sexes.

Proopiomelanocortin mRNA levels were affected by the diet consumed (F_(2,32)_ = 10.8, p < 0.001; **Figure 5C**), with HFD-fed mice having higher POMC mRNA levels compared to the chow and HFDCH groups.

Hypothalamic ObR mRNA levels showed an effect of dietary regimen (F_(2,22)_ = 4.4, p < 0.05; **Figure 5D**), being reduced after HFD and remaining thus in the HFDCH group in both sexes.

**Figure 6:**
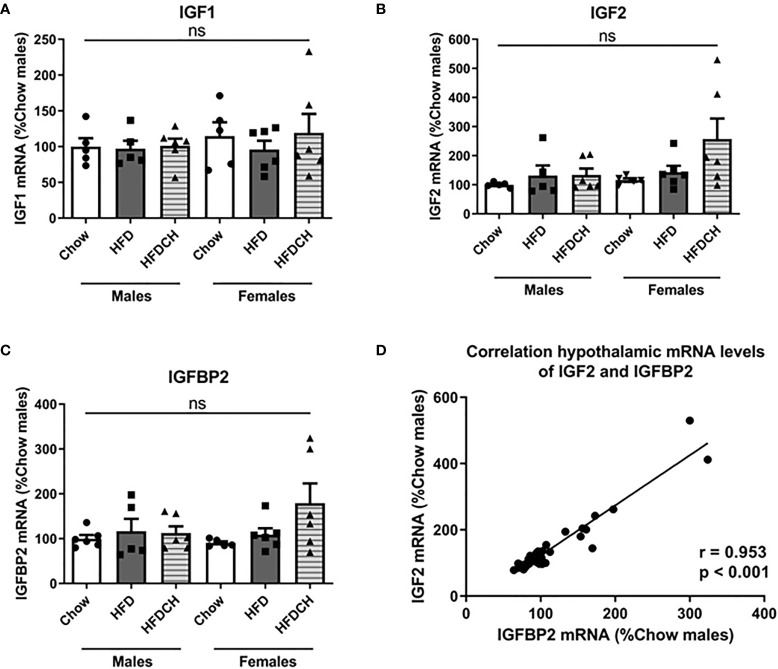
Relative mRNA levels of insulin-like growth factor (IGF)1 **(A)**, IGF2 **(B)** and IGF binding protein (IGFBP)2 **(C)** in the hypothalamus, as well as the correlation of hypothalamic IGF2 and IGFBP2 mRNA levels **(D)**. HFD = high-fat diet (HFD); HFDCH = 8 weeks of HFD followed by 4 weeks of chow. ns, non-significant. n = 6.

Hypothalamic mRNA levels of IGF1 (**Figure 6A**), IGF2 (**Figure 6B**) and IGFBP2 (**Figure 6C**) were not altered by sex or diet. However, as previously reported (10, 11), there was a positive correlation (r = 0.953, p < 0.001) between the hypothalamic mRNA levels of IGF2 and IGFBP2 (**Figure 6D**).

We found no change in response to diet of the protein levels of the glial markers GFAP (**Supplementary Figure 1A**) and Iba1 (**Supplementary Figure 1B**). Likewise, no changes in the mRNA levels of CHOP (**Supplementary Figure 2A**), IL-6 (**Supplementary Figure 2B**) or TNFα mRNA (**Supplementary Figure 2C**) were observed.

FASN mRNA levels showed an effect of the sex (F_(1,23)_ = 16.4, p = 0.001; **Supplementary Figure 2D**), with females having higher levels than males, but with no effect of the dietary regimen. Levels of CPT1A mRNA were not affected by sex or diet (**Supplementary Figure 2E**).

## Discussion

The importance of this study resides in the demonstration that not only do males and females respond differently to HFD-induced weight gain, with significant weight gain being more delayed in females, but also to being returned to a healthy diet. Although females consumed more kilocalories than males on the HFD, as previously described (11, 40), significant weight gain occurred more rapidly in males, with their energy efficiency being higher. Indeed, female mice are more resistant than males to HFD-induced obesity onset (41–43) as well as to obesity-derived metabolic complications such as insulin resistance (44), in part due to the protective effects of estrogens (45, 46). During the later stages of our study females had a relatively greater weight gain on the HFD compared to males, supporting the concept that their response is more delayed, at least in young adult mice.

The preference of mice for HFD over chow has been previously reported (47) and this could possibly explain their drastic reduction in energy intake when returned to a chow diet after consuming the more palatable HFD. As no food choice was available, they then began to consume the chow and their energy intake returned to that of the control group. One month of an *ad libitum* healthy chow diet resulted in weight loss in both sexes, with normalization of body weight occurring more rapidly in females. It was previously reported that in male mice 2 weeks of dietary change from HFD to chow normalized leptin and insulin levels as well as the inflammatory pattern in adipose tissue but body weight and the amount of adipose tissue were only partially recuperated (48), indicating that not all parameters are entirely linked to the normalization of body weight or fat mass. Here in response to a longer period on the chow diet body weight, the percentage of VAT, SCAT and circulating leptin levels were all recuperated, with insulin levels and HOMA also being normalized, but only in males.

Glucose metabolism was affected by HFD in a sex-dependent manner. Although basal glycemia was increased on a HFD and normalized in both sexes after being returned to a chow diet, glucose tolerance was more dramatically affected in males, with the AUC only affected in this sex. This alteration in glucose tolerance was normalized after 3 weeks on a chow diet. A study conducted in male mice exposed to HFD for 56 days reported that GTT and insulin levels were normalized after 9 days on a chow diet (49), suggesting that this parameter may be rapidly normalized by a diet change in male mice. Indeed, Kowalski et al. (49) reported that normalization of the GTT occurred before body weight and energy intake were restored. In contrast, we found that in HFD-induced obese females the GTT AUC was not affected, although both insulin levels and HOMA were altered. Moreover, these latter parameters did not normalize with weight loss. Taking into consideration that glycemia was recovered in HFDCH females but insulin and HOMA-IR were not, suggests that insulin levels increase to maintain a normal glycemia. In a previous study, when female mice were switched from being on a HFD for 12 weeks, where the AUC of the GTT was altered, to chow for 4 weeks body weight, glycemia and AUC of the GTT were all normalized (50). Hence, there are clear sex differences in the alterations of glucose metabolism and the timing of these alterations in response to HFD intake and in response to the return to a healthy diet.

As previously reported in obese patients and mice, circulating levels of free IGF1 (51, 52), total IGF1 (53), IGF2 and IGFBP3 (52) were increased with HFD consumption. These parameters, except for free IGF1, were no longer different from control levels after diet-induced weight loss. This indicates that the IGF system is modified with body weight changes, but diet also affects this system as we previously reported modifications in response to a short-term dietary change that did not affect body weight (10). Thus, a combination of both the type and amount of diet consumed in addition to body weight determines the levels of IGF system members in circulation. Indeed, caloric restriction accompanied by weight loss is also reported to reduce circulating levels of IGF1 in rodents (54–56); however, some studies report an increase in circulating IGFBP3 (55) and others no changes in IGFBP3 (56). In contrast we observed an increase in IGFBP3 after HFD. The differences in the response of IGFBP3 could be due to the variations in the experimental models. Here the reduction in caloric intake was not forced, as they had free access to food, and this protocol was applied to overweight not normal weight mice as in the previous studies.

Some authors have interpreted modifications in circulating IGF2 levels as a prediction of future changes in weight gain (57). We previously found IGF2 levels to be reduced in male rats after one week on a HFD (10) but after 8 weeks of HFD we observed an increase in both male and female mice (11). Here the observation of an increase after HFD and normalization after weight loss contributes to the idea that IGF2 levels are nutritional status dependent.

Circulating IGFBP2 levels are modulated by the metabolic condition and in turn, IGFBP2 can influence metabolism. In obese patients circulating IGFBP2 levels are reduced (52, 58) and increased in patients with anorexia nervosa (58), with a negative correlation with BMI in normal pediatric subjects (58) and adults (59). Mice that over-express IGFBP2 in the liver have a 15-30% reduction in mRNA levels of lipogenic genes such as *Fasn*, with some genes involved in fatty acid oxidation, such as *Ppara*, being increased (59). In mice, weight loss after bariatric surgery increases circulating IGFBP2 levels, and in *Igfbp2*-deficient mice the surgery-induced reduction in adiposity is impaired, as is the early but not long-term improvement in insulin sensitivity and glucose metabolism after surgery (60). In children with obesity the reduced levels of circulating IGFBP2 were found to increase after 6 and 12 months of weight loss although they were not completely normalized (58), most likely because a normal body weight was not yet achieved. Indeed, here both body weight and IGFBP2 levels returned to control levels in male mice with changes in this binding protein only observed in males. A reduction in IGFBP2 has been reported in both male and female patients with obesity (52). The difference with the observations reported here could be due to the time that a subject has been overweight/obese. Female mice were more delayed in developing significant weight gain and some of the associated modifications such as IGFBP2 levels might also be more delayed compared to males, while the patients with changes in this binding protein had more long-term obesity. Moreover, the observation that only males on a HFD had decreased IGFBP2 levels could be related to the different susceptibilities of the sexes to develop both obesity and deregulation of glucose metabolism. We previously found that after 8 weeks of HFD intake circulating IGFBP2 levels were unaffected in male mice (11), while here they were on the HFD for 12 weeks; thus, modifications in IGFBP2 may be delayed in comparison to other parameters. As previously indicated, IGFBP2 is reported to protect against diabetes and obesity onset (37, 38) and here a negative correlation between circulating IGFBP2 and insulin, glycemia, leptin and body weight was found in males. Glucose metabolism was less affected in HFD female mice, and it is possible that modifications in glucose metabolism are required for the decrease in peripheral IGFBP2 levels. As we and others previously demonstrated (10, 61), the peripheral IGF system differs between the sexes, with males having higher total IGF1, IGFBP2 and IGFBP3 and females higher IGF2 levels depending on the diet, with gonadal steroids underlying at least some of these differences (62). Further studies of how sex steroids modify the response of the IGF system to dietary changes would be of great interest, for example how menopause modifies this response.

Hypothalamic neuropeptides regulate energy intake and feeding behavior (30) and long-term HFD consumption led to decreased NPY and AgRP mRNA as previously reported in male (63, 64) and female rodents (11). These modifications may be explained by the satiety properties of HFD, which is more palatable and contains more calories per gram than chow and activates the homeostatic control mechanism to reduce energy intake. Both orexigenic neuropeptides were normalized in the HFDCH mice in males and females, suggesting a return to homeostasis. Leptin, which is elevated in the HFD mice, is transported to the brain and inhibits both NPY and AgRP (65) to decrease food intake. This leptin-mediated inhibition of NPY and AgRP may explain, at least in part, the reduction of these orexigenic neuropeptides after HFD consumption, and their restoration in the HFDCH group where leptin levels are also normalized.

The mRNA levels of NPY and AgRP were higher in females compared to males, as previously reported (66), with this difference being more apparent in chow and HFDCH mice. Female rats are reported to consume a similar amount of palatable food independently of being satiated or hungry, with males adjusting their energy intake to their hunger state (67). The mRNA levels of POMC were increased after HFD and normalized in the HFDCH group in both sexes. Expression of this anorexigenic neuropeptide has been reported to increase in male mice exposed to HFD (64), but also to be unaffected in female (68) and male and female (11) HFD-exposed mice, with the duration of HFD intake possibly being the cause of these discrepancies. Again, the increase in POMC mRNA levels may be due to the higher caloric content of the HFD, promoting satiety and finally contributing to the regulation of energy homeostasis, with the restoration of POMC levels in the HFDCH group reflecting normalization of the energy intake circuitry. Both POMC and NPY/AgRP neurons also modify energy expenditure, which was not analyzed here. Moreover, HFD is reported to have a biphasic effect on locomotor activity in mice depending on the exposition time to HFD with middle-aged female mice having increased locomotor activity after 4 (50) and 5 weeks (69) and in male mice after 3 weeks (70) of HFD intake. When HFD exposition is more long-term the increased locomotor activity decreases (71). Thus, how the reversion to a chow diet affects locomotor activity is of great interest and should be investigated in the future.

Only a few parameters did not normalize after weight loss, including free IGF1. Most studies report that free IGF1 levels increase in obesity (51, 52, 72) as found here, but we found only a partial normalization in the HFDCH groups of both sexes. The increased glycemia associated to obesity is reported to inhibit IGFBP production, resulting in higher free IGF1 levels (73) and accentuating the physiological effects of IGF1 (74). Inversely, a decrease in free IGF1 levels occurs in situations of energy restriction, such as short-term fasting and anorexia nervosa (72). We can speculate that more time at a normal body weight may be needed before a complete restoration of free IGF1 levels is observed.

The changes in serum leptin levels were directly correlated with alterations in adiposity, as might be expected. However, hypothalamic ObR expression levels were decreased in both sexes after HFD, as previously reported in male mice (75), and remained decreased in HFDCH. This may suggest that these mice are less sensitive to the feed-back effect of leptin (76), and this could reflect a potential mechanism by which weight regain may be enhanced if poor dietary habits are restored.

Hypothalamic IGF1 mRNA levels did not change in response to HFD, which contrasts with the increase we previously reported (11, 64). This discrepancy could be due to is the difference in exposition time to HFD, which was 7 or 8 weeks, respectively, in the previous studies as opposed to 12 weeks here. We previously reported that in female mice both hypothalamic IGF2 and IGFBP2 mRNA levels were positively correlated with glycemia (11). Although the peripheral implication of these two factors in the control of glycemia has been reported (25, 38, 77), the involvement of central IGF2 and IGFBP2 in glucose metabolism has been poorly explored. Here hypothalamic IGF2 and IGFBP2 levels were positively correlated, as shown in rats (10) and mice (11). As IGFBP2 has a binding preference for IGF2 compared to IGF1 (39), the connection between these factors in the hypothalamus and their possible involvement in glucose metabolism deserves further investigation.

No evidence of hypothalamic gliosis or inflammation was observed here. Both processes have been reported in mice after long-term HFD intake (78–80) but, other studies described no hypothalamic inflammation after HFD intake in male mice (81) or in either sex (11, 82). Indeed, the causal connection between these phenomena (central inflammation and gliosis) with weight gain remains unclear (83). The exposition time to HFD between these studies and ours differs, which may be related to time dependent changes in central inflammation in response to HFD (78). One possibility is that both the central and peripheral IGFs, that can act as neuroprotective factors and are elevated in HFD may counteract, at least partially, the harmful effects of HFD in the hypothalamus. However, the lack of data concerning the central levels of IGFs in most HFD-induced experiments can only lead us to speculate on this possibility, with more studies being then needed to test this hypothesis.

Here we have shown that HFD-induced overweight mice rapidly return to a normal body weight, with most metabolic parameters returning to control levels when a normal weight and energy intake are restored. This was observed in both males and females although the rate of weight gain and rate recovery differs. Moreover, glucose metabolism and some members of the IGF system in the hypothalamus remained affected in females even after weight recover. Whether the differences between males and females in the modification in glucose metabolism is associated to the sex differences in changes in the IGF system remains to be determined, in addition to determining if the lack of normalization of these factors renders these animals more susceptible to the resumption of inadequate dietary habits

## Data Availability Statement

The raw data supporting the conclusions of this article will be made available by the authors, without undue reservation.

## Ethics Statement

The animal study was reviewed and approved by the Ethical Committee of Animal Experimentation of the Hospital Puerta de Hierro de Madrid and the Animal Welfare Organ of the Comunidad Autónoma de Madrid.

## Author Contributions

Conception and design of study: LF, JA, and JC. Funding acquisition: LF, JA, and JC. Animal handling: SG-C, PR, AF-R, and RC-P. Biochemical analysis: SG-C, SC, and VB. Data discussion and analysis: SG-C, LF, JA, and JC. Redaction of manuscript: SG-C, LMF, and JC. Revision of manuscript: SG-C, LF, SC, PR, AF-R, RC-P, MJ-H, VB, JA, and JC.

## Funding

The authors are funded by grants from the Spanish Ministry of Science and Innovation (BFU2017-82565-C21-R2 to JC & LF), Spanish Ministry of Education, Culture and Sports (university training grant PU13/00909 to AF-R), Fondo de Investigación Sanitaria (PI1600485 and PI1900166 to JA), Fondos FEDER and Centro de Investigación Biomédica en Red Fisiopatología de Obesidad y Nutrición (CIBEROBN), Instituto de Salud Carlos III (JA).

## Conflict of Interest

The authors declare that the research was conducted in the absence of any commercial or financial relationships that could be construed as a potential conflict of interest.

## Publisher’s Note

All claims expressed in this article are solely those of the authors and do not necessarily represent those of their affiliated organizations, or those of the publisher, the editors and the reviewers. Any product that may be evaluated in this article, or claim that may be made by its manufacturer, is not guaranteed or endorsed by the publisher.
