# Supplementary material for: Sex Differences in Metabolic Recuperation After Weight Loss in High Fat Diet-Induced Obese Mice
Source: Front Endocrinol (Lausanne). 2021 Dec 16;12:796661. doi: 10.3389/fendo.2021.796661 (PMC8716724; doi:10.3389/fendo.2021.796661)
Supplement: Supplementary file 1 [file DataSheet_1.pdf]

Supplementary Material

1 Supplementary Data

2 Supplementary Figures and Tables

2.1 Supplementary Figures

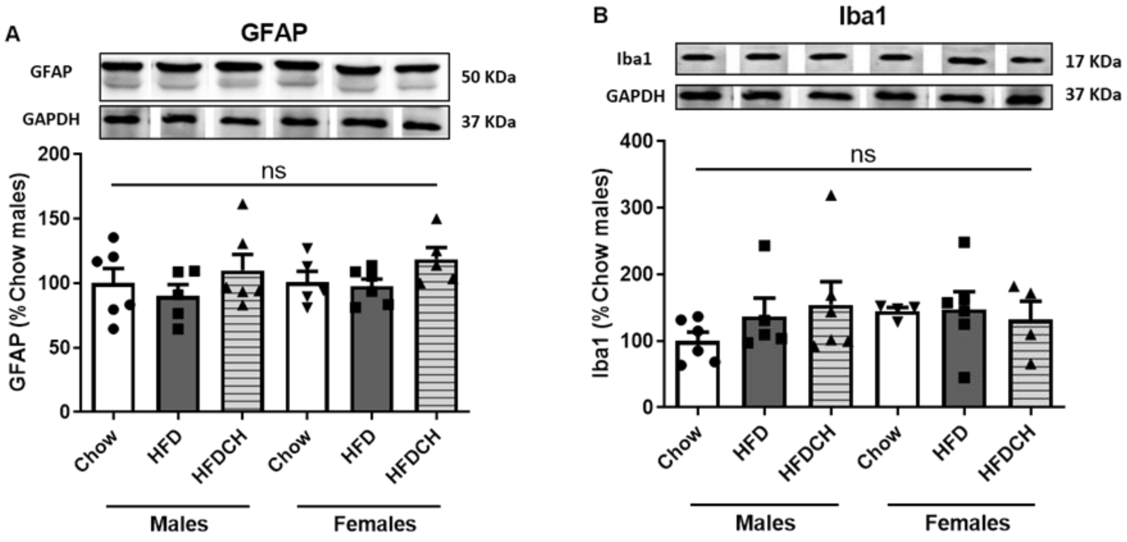

Supplementary Figure 1. Relative hypothalamic levels of GFAP (A) and Iba1 (B) in male and female mice that received chow or a high-fat diet (HFD) for 12 weeks, or HFD for 8 weeks and followed by chow (HFDCH) for 4 weeks. These images are all from the same blot, but were not contiguous and for this reason they are individually placed in order of the experimental groups in the graph. ns = non-significant. n = 6.

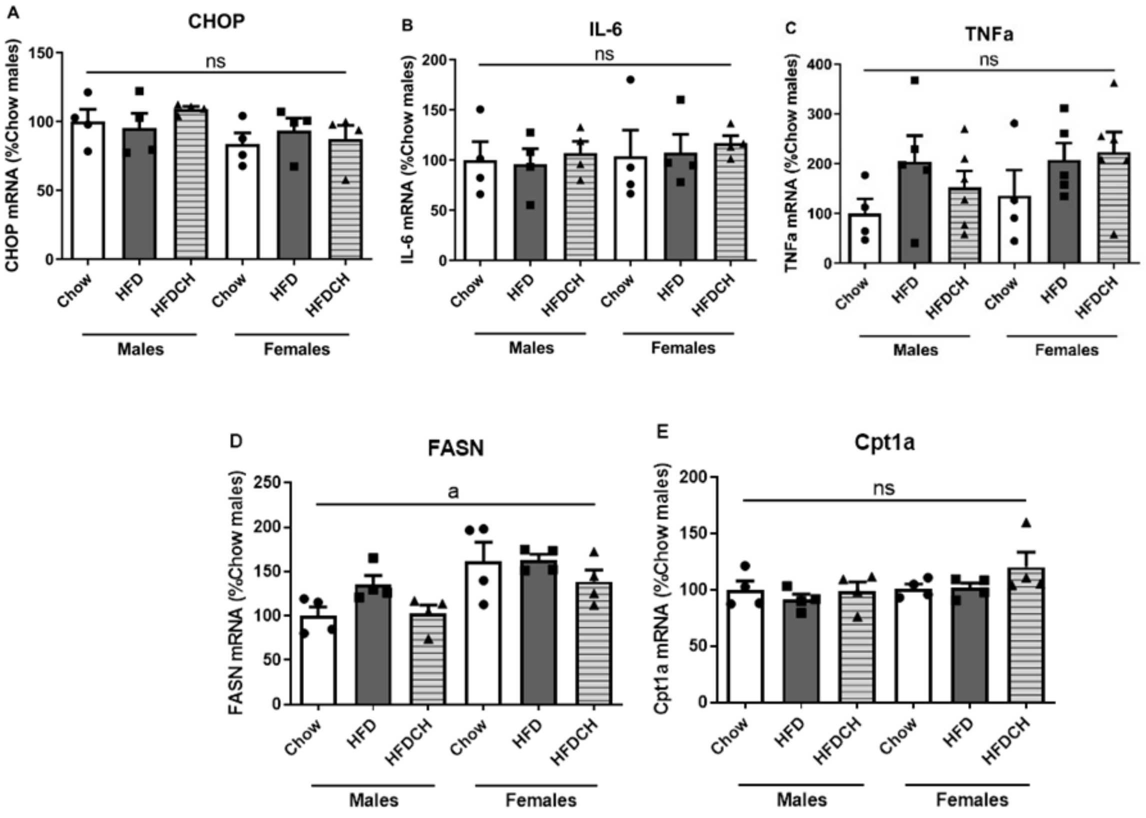

Supplementary Figure 2. Hypothalamic mRNA relative levels of CHOP (A), IL-6 (B), TNFa (C), FASN (D) and Cpt1a (E) in mice which received chow diet or a high-fat diet (HFD) for 12 weeks, or dietary change after 8 weeks of HFD to chow diet during the last 4 weeks (HFDCH). a: effect of the sex, ns = non-significant. n = 4 - 6.
